# Fascin-1 enhances experimental osteosarcoma tumor formation and metastasis and is related to poor patient outcome

**DOI:** 10.1186/s12885-019-5303-3

**Published:** 2019-01-17

**Authors:** Matthias J. Arlt, Aleksandar Kuzmanov, Jess G. Snedeker, Bruno Fuchs, Unai Silvan, Adam A. Sabile

**Affiliations:** 1 Department of Orthopedics, Balgrist Hospital, University of Zürich, Institute for Biomechanics, ETH Zürich, 8008 Zürich, Switzerland; 2 Department of Dermatology, University Hospital Zürich, 8952 Schlieren, Switzerland; 3 Department of Orthopedics and Traumatology, Winterthur Cantonal Hospital, 8401 Winterthur, Switzerland

**Keywords:** Fascin-1, Osteosarcoma, Metastasis, Xenograft mouse model, MMP9

## Abstract

**Background:**

Fascin-1, a prominent actin-bundling protein, is found to be upregulated in several human carcinomas. While it is accepted that Fascin-1 expression correlates with poor clinical outcome and decreased survival in various carcinomas, its role in sarcoma such as osteosarcoma (OS) remains unknown. In the present study, we evaluated the prognostic value and biological relevance of Fascin-1 in OS.

**Methods:**

The correlation between Fascin-1 expression and the outcome of OS patients was determined by immunohistochemistry analysis of Fascin-1 expression in a tissue microarray of OS tissue specimens collected during primary tumor resection. To examine the effect of Fascin-1, shRNA and overexpression technology to alter Fascin-1 levels in OS cells were used in cellular assays as well as in intratibial xenograft OS models in SCID mice.

**Results:**

Kaplan-Meier survival analysis of Fascin-1 expression in OS tumor specimens revealed a direct relationship between Fascin-1 expression and poor patient survival. Furthermore, overexpression of Fascin-1 in OS cells significantly increased their migratory capacity as well as the activity of the matrix metalloprotease MMP-9, known to be critical for the execution of metastasis. Finally, using relevant xenograft mouse models, orthotopic intratibial transplantation of two different OS cell lines overexpressing Fascin-1 promoted tumor growth and lung metastasis.

**Conclusions:**

Collectively, our findings demonstrate for the first time that Fascin-1 has considerable potential as a novel prognostic biomarker in OS, and suggest that targeting of Fascin-1 might be a new anti-metastatic strategy in OS patient treatment.

**Electronic supplementary material:**

The online version of this article (10.1186/s12885-019-5303-3) contains supplementary material, which is available to authorized users.

## Background

Osteosarcoma (OS) is the most frequent primary bone cancer in childhood and has high propensity for metastasis, the major cause of cancer-related death [1, 2]. At the time of diagnosis, 20% of OS patients present detectable metastasis, and in the absence of chemotherapy more than 60% of those with localized disease develop metastases later on [3]. Despite the significant advances in OS treatment, patients with metastases still have a long-term survival rate below 20% [4]. Therefore, identification of novel therapeutic targets remains extremely important to prevent metastasis and treat OS.

Metastasis is a complex multistep process in which tumor cells implement a variety of migratory and invasive strategies in order to infiltrate the primary tissue and disseminate to distant sites. To succeed in cancerous spreading, malignant cells undergo a number of phenotypic adaptations, among which the reorganization of the actin cytoskeleton is critical. It is known that the regulation of the cell cytoskeleton is driven by a collective set of actin binding proteins, which mediate the organization of actin filaments into supramolecular arrangements [5]. A main class of actin binding proteins is the Fascin family, which comprises Fascin-1, − 2, and − 3. Fascin-1, the most ubiquitously expressed family member, regulates adhesion dynamics and crosslinks actin microfilaments into tight and parallel bundles. These bundles are important for the extension of filopodia and invadopodia and for the functionality of these protrusions in promoting cell migration [6–8]. Fascin-1 is absent in most normal epithelia, but is highly upregulated in several human carcinomas, and its expression levels correlate with more aggressive tumors and metastasis [9, 10]. Furthermore, the upregulation of Fascin-1 in a number of carcinomas has been associated with worse clinical outcome, poor prognosis, and shorter survival, indicating that Fascin-1 may play a central role in tumorigenesis and metastasis [11–16]. However, its role in sarcoma and in particular OS has not been described so far. In the present study we have evaluated the prognostic value of Fascin-1 and investigated its regulatory function in OS tumorigenesis and metastasis.

Using a combined immunohistochemistry-based tissue microarray (TMA) and Kaplan-Meier survival analysis with tumor tissue specimens collected from OS patients, we demonstrate that Fascin-1 expression correlates with poor patient survival and can be used as an additional prognostic factor for OS patient outcome. Furthermore, with the aim of investigating its potential regulatory effect, in vitro manipulation of Fascin-1 expression by loss or gain of function demonstrated an important role of Fascin-1 in OS cell motility. In addition, we show that Fascin-1 might facilitate the invasive process by up-regulating MMP-9 activity. Finally, by making use of intratibial xenograft OS mouse models, we showed that overexpression of Fascin-1 in both osteoblastic and osteolytic OS cell lines accelerated the growth of intratibial xenografts and promoted lung metastasis. These results clearly indicate a key role of Fascin-1 in OS progression and metastasis. Altogether, the results reported here point to a great potential of Fascin-1 as a novel prognostic marker as well as a possible target for therapeutic intervention in OS.

## Methods

### Human OS tissue microarray, Fascin-1 immunostaining and Kaplan-Meier analysis

Osteosarcoma and normal bone tissue specimens were collected between June 1990 and December 2005 from 67 patients during primary tumor resection in accordance with the regulations of the local Swiss Ethic committee. The clinical characteristics of the OS patients are presented in Table 1. Among the 67 patients’ cohort, 53 received complete neoadjuvant chemotherapy and the subsequent response was determined histologically on resected tumor specimens according to Salzer-Kuntschik [17]. Salzer-Kuntschik grades I, II, and III were considered to be a good response, whereas grades IV, V, and VI were classified as non-responders. The TMA was constructed and processed as previously described [18, 19], stained with anti-Fascin-1 mouse monoclonal antibody (1400; DAKO), and counterstained with hematoxylin. The grading of Fascin-1 immunostaining was determined based on the intensity and the percentage of the stained area. The percentage of staining was calculated with a custom made MATLAB (v2009b, Mathworks Inc) program as described [20]. Positive stained (brown) and non-stained area (blue) were separated by color deconvolution algorithm [21]. The area percentage of the stain was defined as the positive stained area (number of brown pixels) over total tissue area (number of blue and brown pixels). Grade 1 (non or weak staining) was considered as negative and grades 2 and 3 (moderate and intense staining) were considered as positive for Fascin-1 expression. A Kaplan-Meier analysis was used to correlate Fascin-1 expression with overall survival of the patients.

**Table 1:** Clinical characteristics of Osteosarcoma patients

Patients (*n* = 67)	n	(%)
Gender		
Male	45	67
Female	22	33
Age (years)		
< 10	8	12
10–19	34	51
20–29	15	22
30–39	6	9
≥ 40	4	3
Tumor type		
Osteoblastic	43	64
Chondroblastic	14	21
Fibroblastic	6	9
Telangiectatic	4	6
Anatomic site		
Extremities	57	85
Axial (pelvis or spine)	10	15
Metastasis		
Total metastasis	24	36
Present at diagnosis	5	7
Metastasis after diagnosis	19	28
Chemotherapy response		
Responders (*SK I-III)	30	57
Non-responders (SK IV-VI)	23	43

*SK: Salzer-Kuntschik

### Cell culture, shRNA constructs and retroviral transduction

Human SaOS-2 (ATCC® HTB85™) and 143B (ATCC® CRL-8303™) OS cells, purchased from American Type Culture Collection (ATCC; Manassas, USA), were cultured in DMEM (4.5 g/l glucose)/HamF12 (1:1) medium (Invitrogen) supplemented with 10% heat inactivated fetal bovine serum (FBS, Gibco) at 37 °C in a humidified atmosphere of 5% CO_2_. SaOS-2 and 143B cells were stably transduced with a LacZ gene and selected to obtain SaOS-2-LacZ and 143B-LacZ cell lines as previously reported [22]. SaOS-2-LacZ and 143B-LacZ cells stably over-expressing Fascin-1 (SaOS-2/Fascin-1 and 143B/Fascin-1) were then obtained by transduction with pLenti6/V5-DEST-FASCIN (a gift from Lynda Chin; Addgene plasmid # 31207) and subsequent selection with Blasticidin (Invitrogen, Carlsbad, CA, USA). Control SaOS-2-LacZ or 143B-LacZ cells were obtained by transduction with empty pLentiV5-DEST.

To achieve Fascin-1 silencing, we tested 5 different shRNA sequences cloned into the pLKO.1 retroviral vector (FSCN1 MISSION shRNA; Sigma-Aldrich). After transduction of SaOS-2-LacZ and 143B-LacZ cells, Puromycin resistant cells were selected. The efficiency of Fascin-1 silencing was examined on Western blot, and the most efficient oligonucleotide sequence 5’-CCGGCCCTTGCCTTTCAAACTGGAACTCGAGTTCCAGTTTGAAAGGCAAGGGTTTTTG-3′ was selected and used in the subsequent experiments. pLKO.1 retroviral vector containing a scrambled oligonucleotide sequence was used as control. All generated stable cell lines were authenticated by short tandem repeat DNA profiling (Microsynth) with a PowerPlex®16HS system (Promega) and by comparison with the German Collection of Microorganisms and Cell Cultures database (DSMZ).

### Immunofluorescence and image analysis

Cells grown on glass coverslips were fixed with 3.7% formaldehyde in phosphate-buffered saline (PBS) at room temperature (RT) for 15 min and permeabilized with 0.2% Triton X-100 for 10 min. After incubation at RT for 30 min with blocking solution (0.1% FBS in PBS), the cells were incubated with specific primary antibodies in blocking solution at RT for 1 h. After 3 washing steps with PBS, cells were incubated with fluorescently-labelled secondary antibodies for 45 min at RT. Primary antibodies used were directed against Fascin-1 (1:400; DAKO) and V5 antibody (1:5000; Invitrogen). Filamentous actin was stained by means of Alexa-633-phalloidin and nuclei using NucBlue (Invitrogen). Secondary antibodies were Alexa-Fluor488 conjugated donkey anti-mouse or Alexa-Fluor488 conjugated donkey anti-rabbit (Life Technologies). Immunofluorescent cells were visualized using a spinning disc confocal microscope (iMic, TILL Photonics) equipped with a 60X N.A. 1.35 lens. For the morphological analysis, we used the Cell Profiler software to estimate the perimeter and spreading area of each individual cell [23]. We then used these values to calculate the ratio between them using the formula: R = perimeter * 100 / area.

### Wound-healing migration assay

The migratory properties of OS cells were assessed in a wound healing migration assay. Cells were seeded into 24-well plates. At confluency, wounds measuring between 0.5 and 1 mm in width and approximately 1 cm in length were created with a sterile pin. After 3 washing steps with PBS to remove detached cells and cell debris, the wounds were marked with a Nikon object marker attached to a Nikon Diaphot microscope. Photos of the marked wounds were taken immediately after wounding and after incubation at 37 °C for 16 h. The width of the marked wound areas were measured with the ImageJ Software and the migration rate (μm/h) was then calculated as described [24].

### Zymography and Western blot analysis

The gelatinase activity of MMP2 and MMP9 was analyzed by means of collagen zymography as previously described [25]. For western blot analysis, cells were lysed in ice-cold 50 mM Tris, pH 7.5 containing 250 mM NaCl, 5 mM EDTA, 0.5% NP-40, 50 mM NaF, 0.2 mM Na3VO4, 1 mM dithiothreitol (DTT), 1 mM phenylmethylsulphonyl fluoride (PMSF), and 10 mg/ml aprotinin. Cell lysates were cleared by centrifugation and protein concentrations determined with a Bio-Rad assay (Bio-Rad Laboratories, Hercules, CA, USA). Endogenous and V5-tagged Fascin-1 protein and GAPDH were detected using mouse monoclonal Fascin-1 (1:1000; DAKO), V5 antibody (1:5000; Invitrogen), and rabbit polyclonal anti-GAPDH antibody (1:5000; Santa Cruz Biotechnologies) and corresponding horseradish peroxidase-conjugated secondary antibodies (Santa Cruz Biotechnologies). Peroxidase activity was visualized with the Immobilon chemiluminescence substrate (Millipore, Billerica, MA, USA) and detected with Image Lab System (Bio-Rad, Hercules, CA, USA).

### Human osteosarcoma xenograft mouse model

All animal experiments were performed according to the institutional guidelines and approved by the Ethics Committee of the Veterinary Department, Canton of Zurich, Switzerland. Specific pathogen-free female SCID mice obtained from Charles River (Sulzfeld, Germany) were housed for at least 2 weeks before commencement of the study and then randomly divided into four groups of 12 mice each. As described previously [19], after anesthesia of the mice with a mixture of Fentanyl, Midazolam, and Medetomidin, 10 μl of PBS containing 10^5^ cells was injected into the medullar cavity of the left tibia. The health status of the mice was monitored three times a week and primary tumor growth was monitored weekly by X-ray. The tumor volume was calculated from caliper measurements of the tumor-bearing leg and the healthy control leg by the equation: length × (width)^2^ / 2 of the tumor bearing tibia minus length × (width)^2^ / 2 of the control tibia. At the end of the study, the mice were anaesthetized with a mixture of Ketamine, Xylazine, and Acepromazine. Then, the mice were sacrificed by removal of the blood from the cardiovascular system using PBS perfusion into the right ventricle of the beating heart, and the lungs were prepared. For the LacZ gene expression, the fixed organs were stained with 5-bromo-4-chloro-3-indolyl-β-D-galactoside (X-Gal) solution at 37 °C for at least 3 h as described [22]. Metastases in individual lungs, visible as blue-stained foci, were then quantified by manual counting under the microscope.

#### Statistical analysis

Results of the in vivo experiments are presented as mean ± standard error of the mean (SEM), and quantification results of the Western blots are presented as the mean ± standard deviation (SD). Differences between means were analyzed for significance by unpaired t-test with GraphPad Prism® 5.01 software. The Kaplan–Meier analysis was statistically analyzed with the log-rank test. *p* < 0.05 was considered statistically significant.

## Results

### Fascin-1 expression correlates with poor overall survival of the patients

**Fig. 1:**
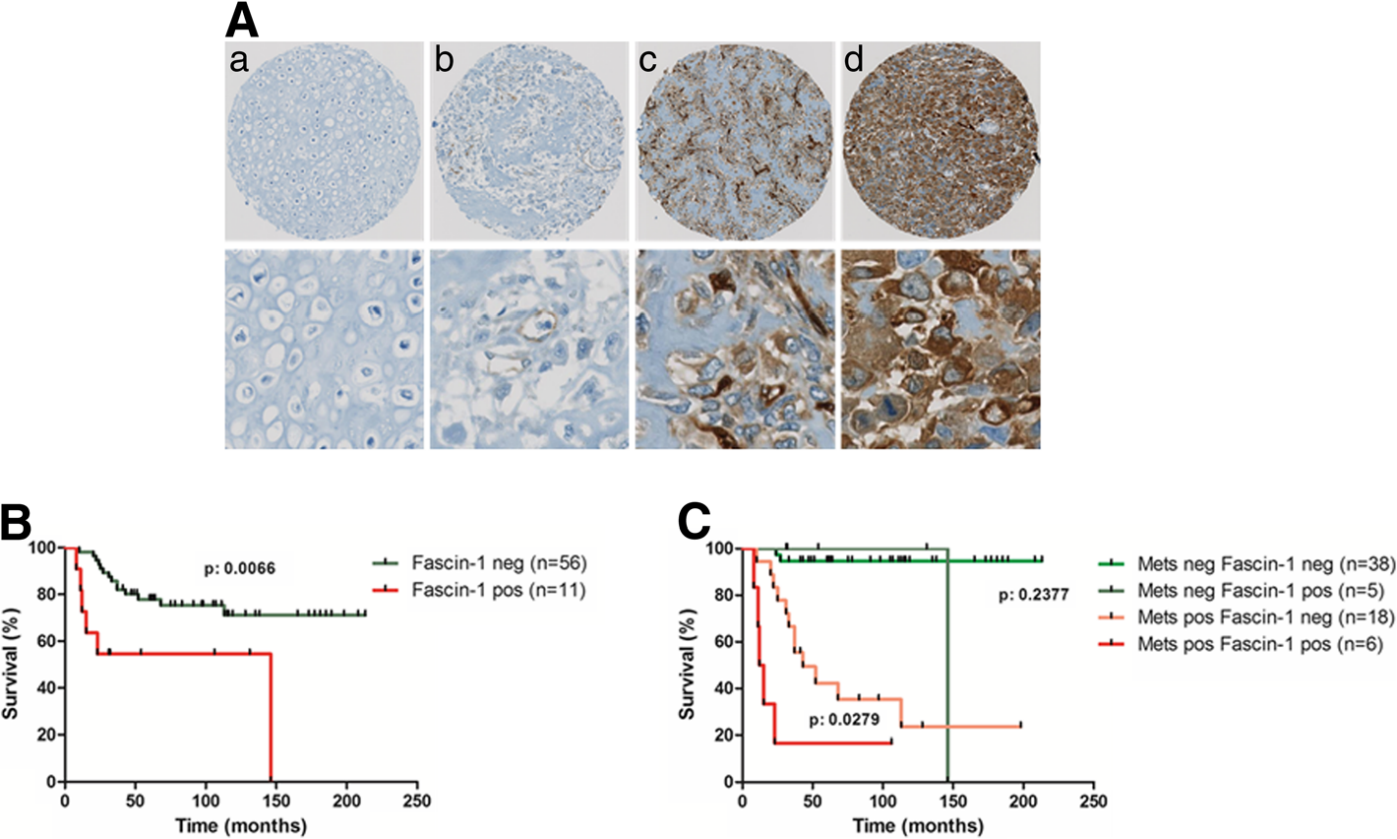
*Kaplan-Meier analysis correlating immunohistochemical staining of Fascin-1 in human OS tissues with overall survival of the patients.* (**A**) Representative images of TMA sections showing entire spots (upper panel) and higher magnification (lower panel) with non-detectable (**a**), weak (**b**), moderate (**c**), and intense (**d**) Fascin-1 immunostaining. (**B**) Overall survival of OS patients with non-detectable (Fascin-1 neg) or detectable (Fascin-1 pos) immunostaining of tumor tissues. (**C**) Overall survival of patients without (Mets neg) or with (Mets pos) metastases and Fascin-1 neg or Fascin-1 pos tumors

To identify effector molecules involved in OS metastasis, we performed combined immunohistochemistry-based TMA and Kaplan-Meier survival analysis. Human OS TMA sections including tumor specimens collected from 67 patients during primary tumor resection were analyzed immunohistochemically for Fascin-1 expression, among them twenty-four patients had metastatic disease (Table 1). Representative images of tissue microarray sections with non-detectable, weak, moderate and intense Fascin-1 immunostaining are presented in Fig. 1a.

A Kaplan-Meier survival analysis showed that, irrespective of local or metastatic disease, patients with Fascin-1 positive expression in tumor tissues had a significantly (*p* < 0.01) shorter overall survival than those with non-detectable Fascin-1 expression (Fig. 1b).

A second Kaplan-Meier survival analysis distinguishing between patients with local or with metastatic disease, revealed that patients with metastases and positive immunostaining for Fascin-1 exhibited a significantly shorter survival than those with metastases and Fascin-1 negative tumors (*p* < 0.05) (Fig. 1c). In the cohort with local disease no significant difference in the survival time was present between patients with Fascin-1 positive tumors and those that were Fascin-1 negative. In a further Kaplan-Meier analysis correlating Fascin-1 expression with the response to neoadjuvant therapy, non-responders who were Fascin-1-positive tended to have a shorter overall survival than Fascin-1-negative non-responders although the difference was not statistically significant (*p* = 0.1069, Additional file 1: Figure S1). Since the lack of statistical significance was likely related to the small number of patients, this finding nevertheless implicate that Fascin-1 might be associated with worse outcome in subsets of OS patients who responded poorly to chemotherapy. All findings taken together imply that the expression of Fascin-1 in OS tumors correlates with a poor prognosis for the patients, and consequently might be considered as new potential predictor for OS outcome.

### Overexpression of Fascin-1 increases cell migration and MMP9 activity

**Fig. 2:**
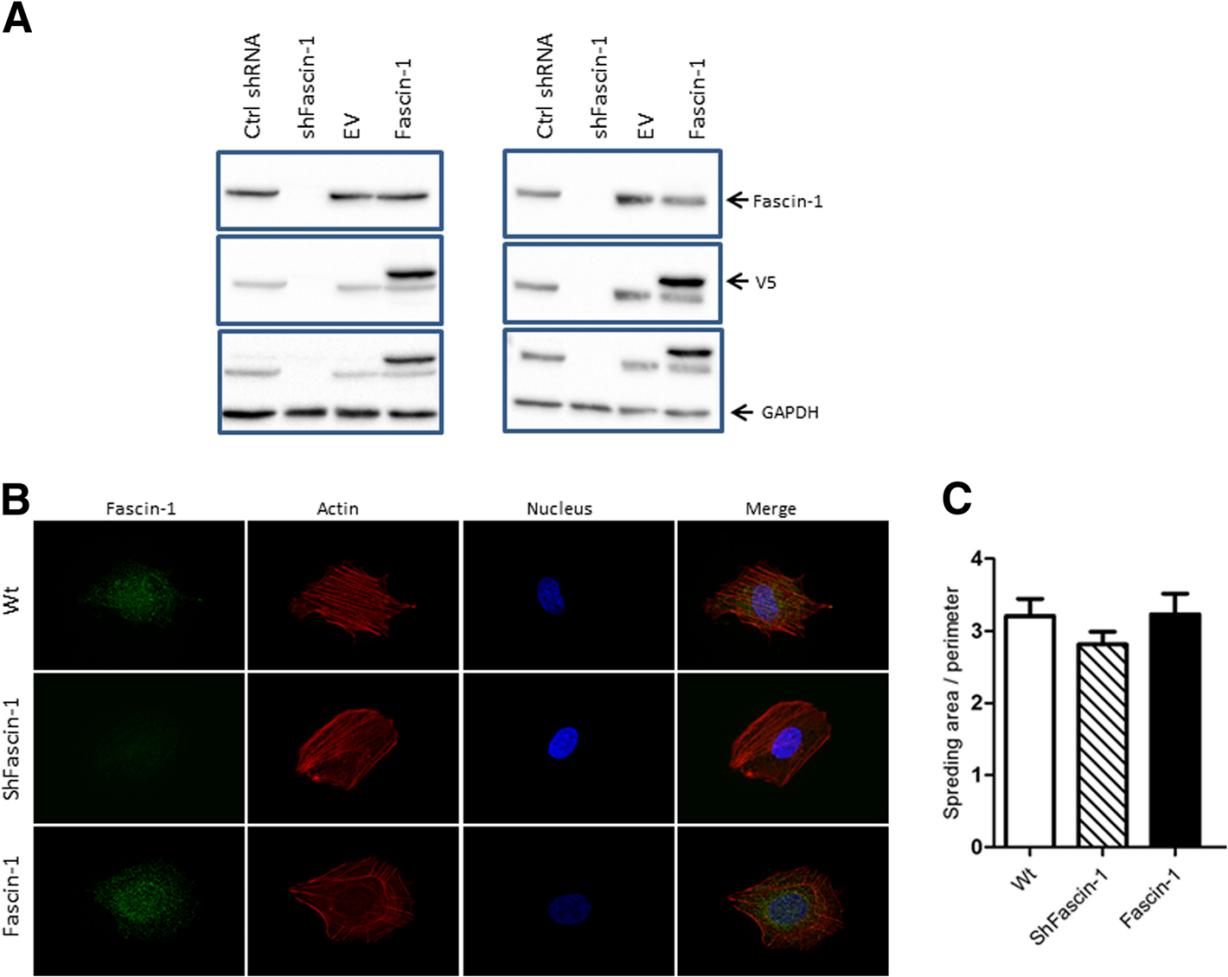
*Validation and characterization of OS cell lines with altered Fascin-1 expression.* (**a**) Western blot analysis with antibodies to Fascin-1 (top panel), to V5 (middle panel), and to GAPDH as a loading control (lower panel) of protein extracts from SaOS-2 (left panel) and 143B (right panel) cells stably transduced with a scrambled control ShRNA (Ctrl ShRNA), a Fascin-1-specific ShRNA (ShFascin-1), a pLenti6/V5-DEST empty vector (EV), or pLenti6/V5-DEST-Fascin-1 (Fascin-1). (**b**) SaOS-2/WT (upper row), SaOS-2/Fascin-1 (middle row), and SaOS-2/ShFascin-1 (bottom row) cells stained with anti-Fascin-1 (green), with Alexa-633-phalloidin (filamentous actin, red), and with NucBlue (nuclei in blue). (**c**) While silencing Fascin-1 reduces the perimeter of the cells in both cases, overexpression has little impact (*n* = 34–57)

In order to assess the function of Fascin-1 in OS and to account for the heterogeneity in phenotypes of OS [26], we used one osteoblastic (SaOS-2) and one osteolytic cell line (143B) and generated from those cell lines stably overexpressing Fascin-1 or with silenced Fascin-1 as described in the methods section. The levels of V5-Fascin-1 expression and the silencing efficiency were determined by western blot (Fig. 2a).

Since Fascin-1 is associated with the initiation and formation of filopodia structures, we considered whether Fascin-1-silencing or overexpression in OS cells may affect filopodia formation. As expected, independently of the type of the cell line, silencing of Fascin-1 was accompanied by a slight decrease in the ratio of cell perimeter by spreading area, suggesting reduced protrusion potential (Fig. 2b and c). Interestingly, Fascin-1 overexpression did not cause detectable differences in comparison to the wild type.

**Fig. 3:**
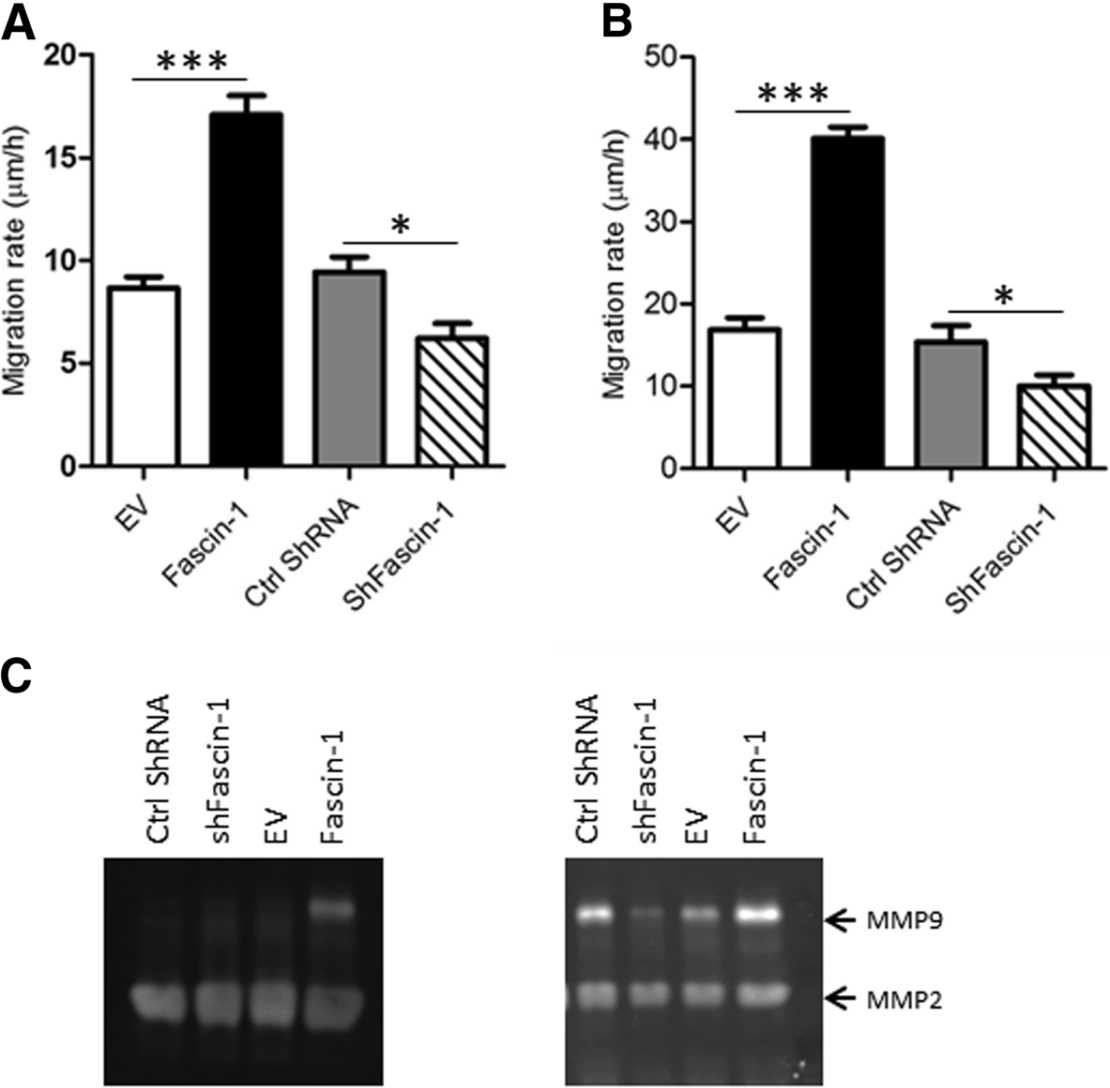
*Migration assessed by wound healing*. Overexpression of Fascin-1 in SaOS-2 (**a**) or in 143B (b) OS cells increases significantly the migration ability. Similarly, silencing of Fascin-1 slightly decreased the migratory rate of SaOS-2 (**a**) and 143B (**b**) cells. Results are the mean ± SEM of at least three independent experiments. (**c**) Zymography analysis showing increased MMP-9 activity in SaOS-2/Fascin-1 (left panel) and in 143B/Fascin-1 (right panel) in comparison to control SaOS-1/EV and 143B/EV cells respectively. Results are the mean ± SEM of three independent experiments

Based on the results of the Kaplan-Meier analyses, we speculated that Fascin-1 expressed by OS tumors may be a stimulatory factor in the regulation of metastatic activity. Consequently, we compared wound-healing migration of SaOS-2 and 143B cells stably overexpressing V5-tagged Fascin-1 (SaOS-2/Fascin-1 and 143B/Fascin-1) with that of empty vector transfected SaOS-2 (SaOS-2/EV) and 143B (143B/EV) control cells with low expression of endogenous Fascin-1. The migration rate of SaOS-2/Fascin-1 cells was 2 times (*p* < 0.01) higher than that of control SaOS-2/EV cells, while silencing of Fascin-1 decreased moderately the migration rate (*p* < 0.05) (Fig. 3a). Similar results were obtained with 143B cells, where Fascin-1 overexpression increased the migration rate by 2.5 folds in comparison to control EV cells (p < 0.01), and silencing of Fascin-1 slightly decreased the migratory rate (p < 0.05) (Fig. 3b). It is noteworthy that Fascin-1 did not affect the proliferation of SaOS-2 and 143B cells in vitro. The calculated doubling times and the percentage of proliferating cells, assessed by immunostaining for the proliferation marker Ki67, were indistinguishable in cultures of cells overexpressing Fascin-1 and control cells (not shown).

Furthermore, we tested whether Fascin-1 enhances the activity of some mediators of metastasis such as MMP-2 and MMP-9, which are among the most well established proteases known to degrade the extracellular matrix and facilitate invasion and metastasis. In contrast to MMP-2, which shows equivalent activity in all studied conditions, up-regulation of Fascin-1 increased significantly the MMP-9 activity compared to the EV control cells (Fig. 3c).

### Fascin-1 enhances intratibial primary tumor growth and pulmonary metastasis in two different xenograft OS mouse models

**Fig. 4:**
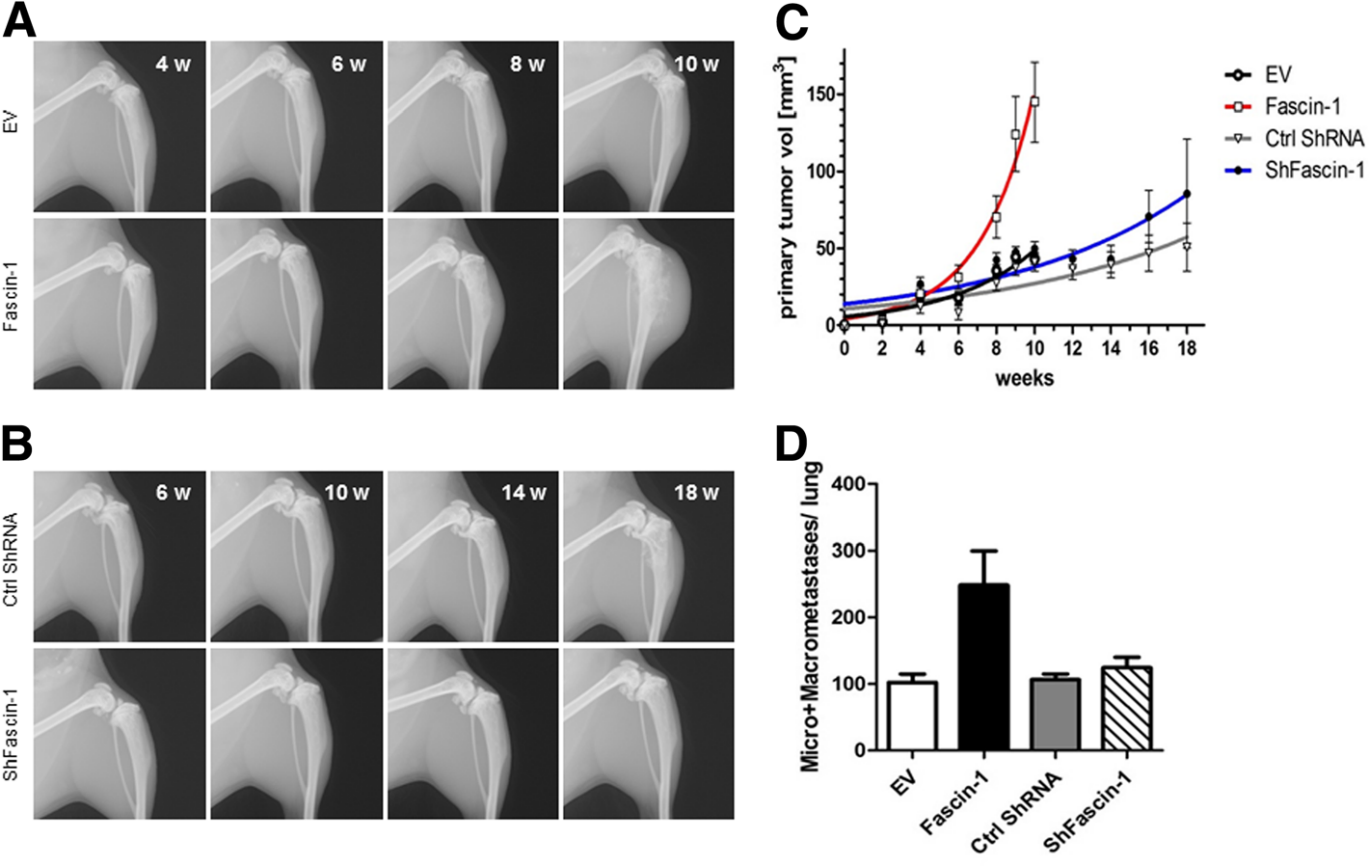
*Fascin-1 overexpression in SaOS-2 cells promotes intratibial primary tumor growth and lung metastasis in SCID mice.* (**a**) Representative X-ray images of tumor-bearing hind limbs of mice injected with SaOS-2/EV cells (upper panel) or with SaOS-2/Fascin-1 (lower panel). The images show primary tumor appearance on indicated days after tumor cell injection. (**b**) Representative X-ray images of tumor-bearing hind limbs of mice injected with SaOS-2/Ctrl ShRNA cells (upper panel) or with SaOS-2/ShFascin-1 cells (lower panel). (**c**) Mean primary tumor growth over time in mice intratibially injected with SaOS-2/EV cells (black), with SaOS-2/Fascin-1 cells (red), with SaOS-2/Ctrl ShRNA (grey) or with SaOS-2/ShFascin-1 blue). (**d**) Mean number ± SEM of metastatic lesions in the lungs

**Fig. 5:**
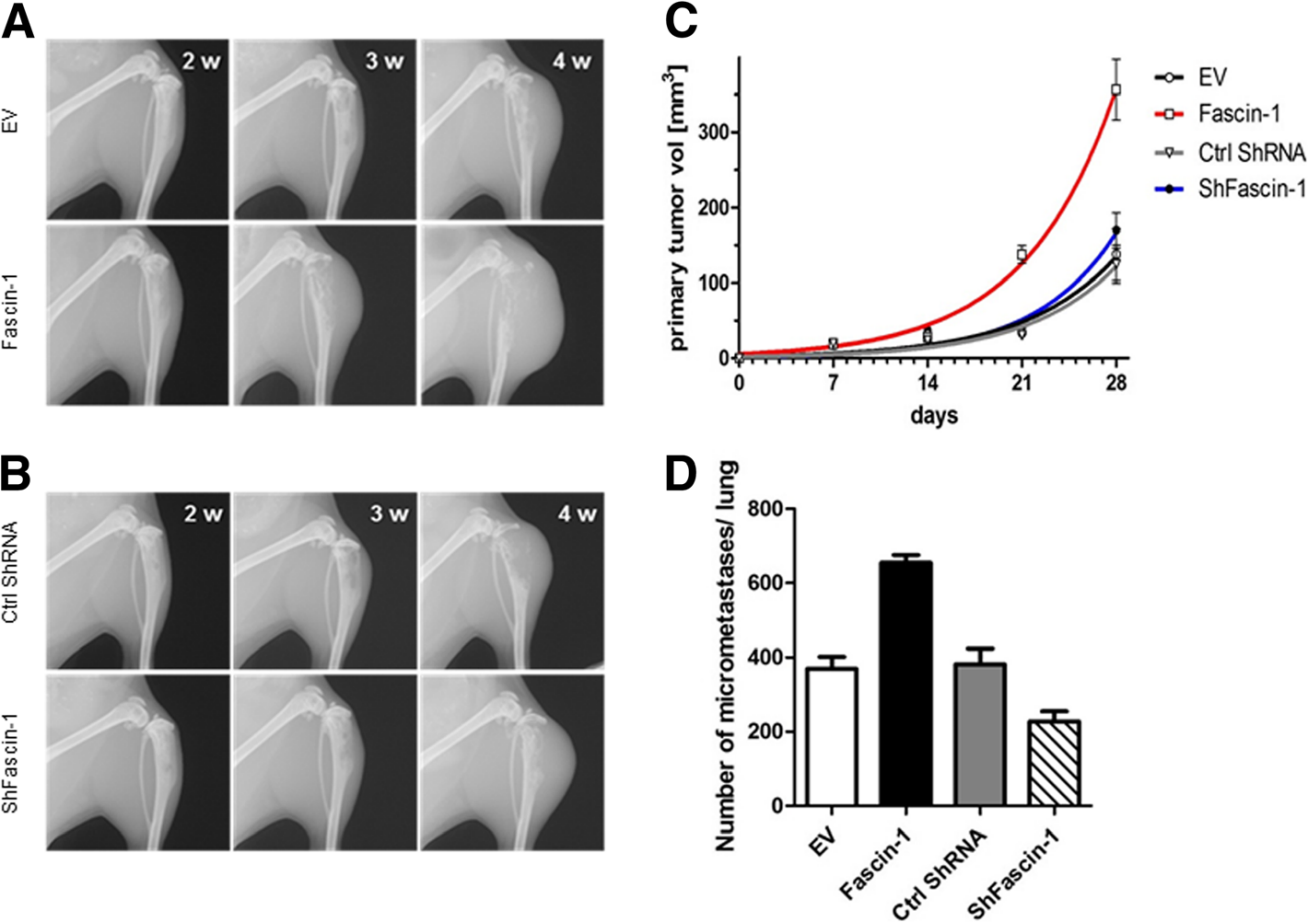
*Fascin-1 overexpression in 143B cells promotes intratibial primary tumor growth and lung metastasis in SCID mice.* **(a)** Representative X-ray images of tumor-bearing hind limbs of mice injected with 143B/EV cells (upper panel) or with 143B/Fascin-1 (lower panel). The images show primary tumor appearance on indicated days after tumor cell injection. **(b)** Representative X-ray images of tumor-bearing hind limbs of mice injected with 143B/Ctrl ShRNA cells (upper panel) or with 143B/ShFascin-1 cells (lower panel). **(c)** Mean primary tumor growth over time in mice intratibially injected with 143B/EV cells (black), with 143B/Fascin-1 cells (red), with 143B/Ctrl ShRNA (grey) or with 143B/ShFascin-1 blue). **(d)** Quantification of the number of metastatic lesions in lungs

To study the impact of Fascin-1 on tumor growth and spreading in a physiologically faithful microenvironment, we performed orthotopic transplantation of OS cell lines into the tibia of SCID mice. Orthotopic tumors derived from injected SaOS-2 cells are mainly osteoblastic, resembling the osteogenic tumor phenotype in humans [19, 27], while, upon intratibial injection of 143B cells, the resulting primary tumors are predominantly osteolytic, thereby representing the osteolytic OS cases seen in human patients [28]. Irrespectively of the phenotype of the used OS cells, the generated experimental tumors preferentially metastasize in lungs. Thus, these orthotopic mouse models reflect more closely the clinical pattern than intravenous injection or subcutaneous implantation of OS tumor cells [29]. To assess the effect of Fascin-1 on OS malignancy we compared the tumorigenic and metastatic potential of SaOS-2-LacZ and 143B-LacZ cells over- and under-expressing Fascin-1. As shown in Fig. 4, mice injected with SaOS-2/Fascin-1 cells developed fast growing primary tumors six weeks after injection and showed osteoblastic lesions in the bone and the surrounding soft tissue (Fig. 4a, lower panel). In contrast, in mice injected with SaOS-2/EV cells, primary tumors remained radiologically undetectable until week eight (Fig. 4a, upper panel). Mice of both SaOS-2/Fascin-1 and SaOS-2/EV groups were sacrificed after 10 weeks because the animals in the SaOS-2/Fascin-1 group became moribund. Accordingly to the X-ray monitoring, Fascin-1 xenografts with a final mean primary tumor volume of 145 ± 25.8 mm^3^ (SaOS-2/Fascin-1) were significantly (*p* < 0.05) larger than SaOS-2/EV (43.2 ± 3.5 mm^3^) xenografts (Fig. 4c). Similarly, the calculated mean primary tumor volume (356.4 ± 40.1 mm^3^) of 143B/Fascin-1 xenografts were significantly (p < 0.05) bigger (137.8 ± 34.6 mm^3^) than 143B/EV xenografts (Fig. 5a and c). Moreover, the ex vivo X-gal-staining of lung whole mounts revealed that SaOS-2/Fascin-1 and 143B/Fascin-1 cells generated an average number of metastatic lesions 2.5 and 2 times larger than wild type cells respectively (Fig. 4d and 5d). It is noteworthy that silencing of Fascin-1 in both SaOS-2 and 143B cell line systems did not significantly affect neither primary tumor growth (Fig. 4b/c and Fig. 5b/c respectively), nor metastasis formation (Fig. 4d and 5d respectively). However, although not statistically significant, the metastatic potential of ShFascin-1 in 143B cells was nearly 40% lower than control cell (Fig. 5d). Taken together, our data demonstrates for the first time that Fascin-1 enhances primary tumor formation and promotes lung metastasis in OS.

## Discussion

Our study demonstrates that Fascin-1 expression significantly correlates with OS progression and metastasis, suggesting Fascin-1 as a potential prognostic biomarker for poor outcome of OS patients.

Osteosarcoma is the most frequent primary bone cancer in childhood with high propensity for metastasis, the major cause of cancer-related death [1, 2]. Therefore, understanding the molecular basis of OS progression, especially during invasion and metastasis, and the identification of potential prognostic and predictive biomarkers and therapeutic targets for OS treatment are required to refine treatment and to improve the currently poor patient outcome. In an effort to identify valuable regulatory biomarkers, we used high-throughput tissue microarray and immunohistochemistry with antibodies against a panel of regulatory molecules. With this approach, we identified and selected Fascin-1 for further investigation. Indeed, it is known that the acquisition of invasive and metastatic potential of tumor cells is associated with the reorganization of the actin cytoskeleton. In this regard, Fascin-1, an actin binding protein that crosslinks actin filaments into bundles appears up-regulated in some carcinomas and has been previously proposed as a therapeutic biomarker [10, 16, 30, 31]. However, the biological relevance of Fascin-1 expression in OS progression and metastasis is not reported up to date. Therefore, we have explored Fascin-1 as a potential prognostic marker for OS.

The Kaplan-Meier survival analysis revealed a significant correlation between Fascin-1 expression and poor patient prognosis. Furthermore, the analysis confirmed the well-documented clinical observation that mortality of OS patients is manly caused by metastasis of the primary tumor. However, our data also showed that Fascin-1 expression in primary tumors defines a subgroup within the metastatic disease cohort with significantly worse prognosis, indicating an important role of Fascin-1 in this life-threatening step of OS progression. These findings are in line with the results reported for carcinomas [10, 32–34].

Furthermore, we overexpressed and silenced the expression of Fascin-1 gene in SaOS-2 and 143B cells and studied their impact in vitro and in vivo in an intratibial xenograft OS model in SCID mice. Because Fascin-1 is best known for its function as actin cytoskeleton organizer, we first investigated its impact on actin-related processes, namely, cell morphology and motility. Image analysis of wild type and OS cells with silenced or overexpressed Fascin-1, revealed that while silencing of Fascin-1 does reduce the amount of membrane protrusions, overexpression does not have a significant impact on cell shape. Because Fascin-1 requires post-translational modifications for its filopodial-stabilization function, it is possible that in OS cells overexpression of Fascin-1 is insufficient to cause a significant increase in these protrusions [35, 36]. In turn, wound healing assays revealed increased migration velocities in OS cells overexpressing Fascin-1, while the impact of Fascin-1 repression was not significant.

Besides its structural functions, Fascin-1 is as well known to participate in other cellular processes, including transcriptional regulation [37]. In this regard, our experiments revealed that forced expression of Fascin-1 in OS cell lines enhances MMP9 secretion and activity. Because augmented cell motility and protease activity contribute to the cascade that leads to metastasis, these findings underscore the importance of Fascin-1 expression in the malignant progression of OS, and are in line with the results reported for other cancers [10, 34, 38, 39].

Our study also revealed that OS cells overexpressing Fascin-1 show a markedly enhanced in vivo malignant phenotype compared to control cells in intratibial xenograft OS mouse models. This was evidenced by accelerated primary tumor growth, and higher percentage of metastatic lesions of the mice injected with cells overexpressing Fascin-1 compared with the animals that received control cells. These observations are in good agreement with the results reported for carcinomas [40, 41]. However, despite the strong effect of Fascin-1 overexpression, silencing of Fascin-1 did affect neither primary tumor formation, nor metastasis formation in our intratibial xenograft models. This finding is different to the results described in carcinomas such as colon adenocarcinoma or esophageal squamous cell carcinoma. When invasiveness of cells isolated from these epithelial tumors were analyzed in mouse xenograft models, silencing of Fascin-1 results in decreased primary tumor growth [42–44]. It is however noteworthy that these animal studies employed heterotopic xenograft mouse models with subcutaneous cell inoculation, as opposed to our in vivo study in which we performed orthotopic (intratibial) tumor cell injection, which takes into account the tumor microenvironment. It is well-documented that the dynamic crosstalk between tumor cells and the local microenvironment critically influences their ability for migration, invasion, and metastasis [45]. Otherwise, as it is well acknowledged that functionally related proteins cooperate to achieve their function, one could speculate that some other factors might functionally interact with Fascin-1, and thereby Fascin-1 silencing could be compensated by these proteins. Indeed, the actin bundling factor Daam1 and L-plastin were shown to functionally cooperate with Fascin-1 in the stabilization of invadopodia and filopodia in cancer cells [46, 47]. The fact that Fascin-1 homozygous KO mice are viable and fertile [48], further supports the idea that other factors compensate the absence of Fascin-1.

## Conclusions

In conclusion, we show for the first time that Fascin-1 expression significantly correlates with OS progression and metastasis, revealing Fascin-1 as a potential prognostic biomarker for poor outcome of OS patients. Our data provide a rationale for future clinical investigations of Fascin-1 as a therapeutic target in OS.

## Additional file

Additional file 1: **Figure S1.** *Kaplan-Meier analysis correlating the overall survival of responders and non-responders patients to neoadjuvant therapy with Fascin-1 immunohistochemical staining.* The response to the therapy was determined histologically on resected tumor specimens according to Salzer-Kuntschik, and both responders and non-responders patients are included in the analysis. To evaluate the relevance of our patient cohort, we determined the correlation of chemotherapy response and the presence of metastases with the overall survival of the patients, since these are known as key prognosis indicators in OS. As expected, non-responders and metastases-positive patients had significantly shorter overall survival than responders and metastases-free patients (not shown). Furthermore, we determined the correlation of the overall survival of non-responders and responders to neoadjuvant therapy with Fascin-1 staining. As shown in Fig. S1, we did not observe any significant difference in survival between Fascin-positive and Fascin-negative non-responders patients. Kaplan-Meier analysis correlating immunohistochemical staining of Fascin-1 in human OS tissues with overall survival of non-responders and responders to neoadjuvant therapy. This analysis excluded specimens from patients who didn’t receive neoadjuvant therapy (*n* = 3) or received incomplete therapy (*n* = 10). (PNG 66 kb)

## Abbreviations

MMP: Matrix metalloproteinase
OS: Osteosarcoma
TMA: Tissue microarray
