# Supplementary figures and images for: Fascin-1 enhances experimental osteosarcoma tumor formation and metastasis and is related to poor patient outcome
Source: BMC Cancer. 2019 Jan 17;19:83. doi: 10.1186/s12885-019-5303-3 (PMC6337773; doi:10.1186/s12885-019-5303-3)

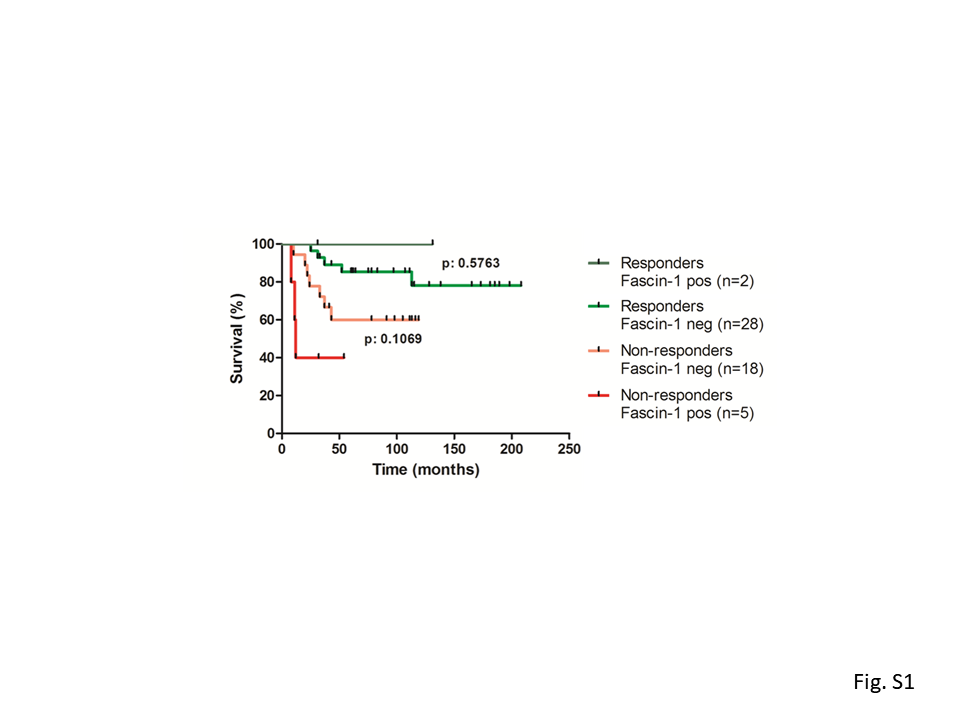

Supplement: Supplementary file 1 — Figure S1. Kaplan-Meier analysis correlating the overall survival of responders and non-responders patients to neoadjuvant therapy with Fascin-1 immunohistochemical staining. The response to the therapy was determined histologically on resected tumor specimens according to Salzer-Kuntschik, and both responders and non-responders patients are included in the analysis. To evaluate the relevance of our patient cohort, we determined the correlation of chemotherapy response and the presence of metastases with the overall survival of the patients, since these are known as key prognosis indicators in OS. As expected, non-responders and metastases-positive patients had significantly shorter overall survival than responders and metastases-free patients (not shown). Furthermore, we determined the correlation of the overall survival of non-responders and responders to neoadjuvant therapy with Fascin-1 staining. As shown in Fig. S1, we did not observe any significant difference in survival between Fascin-positive and Fascin-negative non-responders patients. Kaplan-Meier analysis correlating immunohistochemical staining of Fascin-1 in human OS tissues with overall survival of non-responders and responders to neoadjuvant therapy. This analysis excluded specimens from patients who didn’t receive neoadjuvant therapy (n = 3) or received incomplete therapy (n = 10). (PNG 66 kb) [file 12885_2019_5303_MOESM1_ESM.png]
